# Postoperative Cognitive Dysfunction

**DOI:** 10.5005/jp-journals-10071-23196

**Published:** 2019-06

**Authors:** Indu Kapoor, Hemanshu Prabhakar, Charu Mahajan

**Affiliations:** 1-3 Department of Neuroanesthesiology and Critical Care, AIIMS, Delhi, India

**Keywords:** Anesthesia, Cognitive dysfunction, Neurocritical care, Postoperative period

## Abstract

Cognitive dysfunction is a common complication in primary or metastatic brain tumors and can be correlated to disease itself or various treatment modalities. The symptoms of cognitive deficits may include problems with memory, attention and information processing. Primary brain tumors are highly associated with neurocognitive deficit and poor quality of life. This review discusses the pathophysiology, risk factors and assessment of cognitive dysfunction. It also gives an overview of the effect of anesthetics on postoperative cognitive dysfunction and its management.

## INTRODUCTION

Cognitive dysfunction is a common complication in primary or metastatic brain tumors and can be correlated to disease itself or various treatment modalities. The symptoms of cognitive deficits may include problems with memory, attention and information processing. Primary brain tumors are highly associated with neurocognitive deficit and poor quality of life. There are number of studies indicating the association of brain tumors and their treatment modalities to the cognitive dysfunction.^1,2^ Cognitive function can also be related to tumor laterality. Patients with tumors in left hemisphere will have lower scores on verbal tests, whereas tumor in right hemisphere will have lower scores on facial recognition tests.^3^ Patients with tumors in the left hemisphere report more difficulty in concentrating and those with right-hemisphere lesions,report more tension.^4^ Since most of the patients of brain tumor cannot be cured with surgery alone, improvement of palliative care of the symptoms and cognitive function are important part of the treatment. Now the cognitive function has also been considered as an independent prognostic factor in the survival of patients of brain tumor.^5^ Compared to patients with non-central nervous system tumors, brain tumor patients report more fatigue, cognitive dysfunction, and altered mood states.^6^ In contrast, cognitive dysfunction in patients undergoing spine surgeries have not been studied widely. Fewer studies have shown cognitive impairment in patients who underwent elective cervical and lumbar spine surgery.^7^ Patients with traumatic brain injury may experience cognitive dysfunction in the first hours following the trauma.^8,9^ It has been observed that patients with traumatic brain injury experience worse cognitive outcome compared to patients with orthopedic injury and other injuries.^10^^,11^ Postoperative cognitive dysfunction is also encountered after cardiac surgery, non-cardiac surgery and even with procedures under sedation.^12^

## INCIDENCE

Savageau first described an association between cognitive dysfunction, surgery and anesthesia exposure in 1982.^13^ As per the International Study of Postoperative Cognitive Dysfunction (ISPOCD), the prevalence and risk factors associated with POCD in the elderly population (mean age 68 years, range 60–81 years) is found to be 26% in fist week postsurgery with 10% of patients exhibiting POCD 3 months postsurgery. The age along with duration of anesthesia and poor education were significant risk factors in the development of POCD.^14^ The highest incidence of POCD is found in elderly patients,^8^ but it can develop in all age groups.

## PATHOGENESIS

Numerous neurotransmitters like norepinephrine, lymphokines, melatonin have been implicated in the pathogenesis of POCD.^15,16^ Chemokines scan cause disruption of blood brain barrier *in vitro*, and suggested to be associated with pathogenesis of delirium. Its level has been found to be elevated in early postoperative period in patients who develop delirium after surgery.^17^

## RISK FACTORS

Risk factors leading to POCD can be divided in to preoperative, intraoperative and postoperative factors (Table 1). Several precipitating factors which can aggravate the symptoms of POCD include: sleep deprivation, Foley's catheterization, multiple medications, central venous line insertion, infection and pain. Good patient staff communication, appropriatepatient care and sanitization can prevent the inclination of POCD symptoms in such patients.

## COGNITIVE ASSESSMENT

The cognitive function evaluation has always been a grey area for many clinicians, as cognitive assessment is not included in routine examination in patients. It would be beneficial for patients to prevent the development of common cognitive disorders such as dementia, delirium, and postoperative cognitive dysfunction. Cognitive evaluation in difficult in patients specifically neurosurgical patients with impaired mental status. Cognitive status should be evaluated at two stages: first during preoperative period particularly in elderly patients in a way to identify postoperative cognitive dysfunction and next tests should be done during post- operative period to detect any immediate and late changes. One should also follow up the patients with high risk of developing cognitive dysfunction even after 6 months or longer.

**Table 1:** Risk Factors for POCD

*Preoperative factors*	*Intraoperative factors*	*Postoperative factors*
Age >70 years	Severe bleeding (>1000 mL)	Severe pain
Orthopedic, abdominal aortic aneurysm, and cardiac thoracic surgery	Intraoperative tight glucose control	Benzodiazepine and anticholinergic drugs
History of alcohol or illicit drug abuse	Bispectral index (too low and too high)	Delayed ambulation
Electrolyte abnormalities	Intraoperative hypotension and hypocapnia	Malnutrition
Alzheimer's disease (AD)		

Neuropsychologists should be approached for complete and detailed assessment of patients suspected of have cognitive impairment. There are number of tests that could be done in postoperative period to assess the cognitive impairment. In immediate postoperative period consciousness assessment is done using Glasgow coma scale (GCS) and Riker agitation sedation scale (RASS). If RASS is >3, presence of delirium will be assessed with confusion assessment method short version (CAM-ICU) and nursing delirium screening scale (NU-DESE). Cognitive function will be assessed with battery of tests like Mini-mental state examination (MMSE), digital symbol substitute test, trail making test (TMT), stroopcolor word interference test, Clock Drawing Test and memory impairment screen (MIS). Postoperative memory can be assessed by Galveston orientation and amnesia test (GOAT) and digit span test (DST).

There is no gold standard tests to measure cognitive function, but the combination of tests would help in diagnosis of cognitive impairment and to strategize for prevention of further impairment that would benefit in the overall outcome of patient.

## ANESTHETIC EFFECTS

Apart from primary brain lesions, other factors which could lead to postoperative cognitive deficit are surgery,^1^ radiotherapy, chemotherapy, antiepileptics, corticosteroids and various anesthetic agents.^18,19^ The literature is scarce on the severity, incidence and effect of anesthetics on cognition function of these patients.Fridrikson et al conducted an animal study in neonatal mouse brain and observed reduction in cognitive function , after a combination of thiopental or propofol and ketamine at postnatal day 10 and at 8–10 weeks of age.^20^ Postoperative memory impairment have been observed in patients who have received 1–2 hours of propofol and remifentanil based anesthesia and associated reduced psychomotor function up to the first postoperative day.^21^ On comparison, propofol has been found to provide better cognition scores compared to sevoflurane than isoflurane in patients undergoing transsphenoidal resection of pituitary tumors.^19^ However another study found no different in early cognitive function between sevoflurane and propofolin patients undergoing craniotomy for supratentorial intracranial surgery.^22^ No differences in the incidence of post operative cognitive dysfunction has been demonstrated in patients anesthetized with either xenon, propofol, desflurane, or sevoflurane.^23-25^ Opioids and associated disturbances of calcium, sodium, and glucose homeostasis has been shown to cause post operative cognitive dysfunction.^26^ There have been conflicting results on literature search regarding impact of inhalational anesthetic agents on POCD. A systemic review of its kind by Zou YQ et al to assess the effect of different inhalational anesthetics on postoperative cognitive function is under protocol stage and is expected to guide us on effect of various anesthetic agents on cognitive functions.^27^ Though the exact pathogenesis of POCD is not simple and is multifactorial, it has been shown to be associated with amyloid beta deposition and astrocytic gliosis like in Alzheimer disease.^28^ Animal studies have shown that anesthetic sparticulary inhalational agents can increase the development of these pathological markers in the brain.^29,30^ Xenon an insert gas with an ideal anesthetic property to be used in neurosurgical patients have not been shown to cause delirium or cognitive dysfunction in elderly patients.^31^

## MANAGEMENT

At present there is no specific treatment for postoperative cognitive dysfunction. Though patients receive symptomatic treatment, the overall outcome could be variable ranging from short term cognitive dysfunction to long term cognitive decline. The pathogenesis of POCD is poorly understood and that has necessitated the large clinical trials and new interventions to prevent this catastrophic complication particularly in elderly patients who are at higher risk compared to other patients.

## RECENT LITERATURE

According to a recent Cochrane database of systematic reviews, it is not clear whether maintenance with propofol-based total intravenous anesthesia (TIVA) or with inhalational agents affect incidences of postoperative delirium, mortality, or length of hospital stay. Low certainty evidence found that maintenance with propofol-based TIVA may reduce POCD.^32^ Numerous ongoing studies have been observed from clinical trials register searches. Results from these studies in future may provide more certainty for clinical outcomes. Moderate-quality evidence suggest that optimized anesthesia guided by processed electroencephalographic (EEG) indices could reduce the risk of postoperative delirium in patients aged 60 years or over undergoing non-cardiac surgical and non-neurosurgical procedures. There are no data available for patients under 60 years.^33^

## CONCLUSION

With an advent of newer neurosurgical techniques and pharmacological drugs, life expectancy is on increasing trend. That has surely lead to an increase in the number of elderly patients exposed to surgery and anesthetic drugs. Thus it is of utmost importance to understand the effect of them on cognitive function. So to optimize the care and to prevent the occurrence and aggravation of symptoms of POCD, clinicians should be familiar with the condition called postoperative cognitive dysfunction, its assessment, prevention and treatment.
